# Are Métis mothers receiving adequate prenatal care in Ontario? A population-based study

**DOI:** 10.23889/ijpds.v9i5.2542

**Published:** 2024-09-18

**Authors:** Helana Boutros, Noel Tsui, Haley Golding, Abigail Simms, Sarah Edwards

**Affiliations:** 1 Métis Nation of Ontario; 2 ICES Central

## Objective and Approach

In Canada, Métis people are one of three distinct Indigenous Peoples whose rights are recognized and affirmed in Section 35 of the federal Constitution Act, 1982. Currently, there is a paucity of health research regarding Métis maternal and prenatal in Canada. Prenatal care is a frequently used health service that can reduce the incidence of perinatal morbidity and mortality and measures of adequate prenatal care have been established in the Canadian context. The objective of our study is to examine the receipt of adequate prenatal care among mothers who are citizens of the Métis Nation of Ontario (MNO) who had a live birth between 2012 and 2022. This population-based study will use the MNO citizenship registry linked to health administrative and demographic databases in Ontario, Canada’s most populous province.

## Results and Conclusion

There were 2,615 live births to Métis mothers registered with the MNO between 2012 and 2022. Métis mothers were younger and more likely to live in a rural location relative to other Ontarian mothers. Analysis is still ongoing and results on prenatal care adequacy will be presented.

## Implications

The findings may be used to inform MNO’s Healthy Babies Healthy Children program, designed to support Métis mothers, children, and families from prenatal to six years of age across Ontario. The program’s goal is to ensure equitable access to resources, support, and knowledge for Métis families, so their children have the best possible opportunity to thrive.

